# Susceptibility of schizophrenia and affective disorder not associated with loci on chromosome 6q in Han Chinese population

**DOI:** 10.1186/1744-9081-3-46

**Published:** 2007-09-14

**Authors:** Zuowei Wang, Yiru Fang, Shunying Yu, Chengmei Yuan, Wu Hong, Zhenghui Yi, Sanduo Jiang, R Kelsoe John, Zucheng Wang

**Affiliations:** 1 Department of Psychiatry, School of Medicine, Shanghai JiaoTong University, Shanghai, PRoC; 2 Shanghai Mental Health Center, 600 South Wan Ping Road, Shanghai, PRoC; 3 Hongkou Mental Health Center of Shanghai, 159 Tongxin Road, Shanghai, PRoC; 4 Departments of Psychiatry, University of California, San Diego, and San Diego VA Healthcare System, La Jolla, CA, USA

## Abstract

**Background:**

Several linkage studies across multiple population groups provide convergent support for susceptibility loci for schizophrenia – and, more recently, for affective disorder – on chromosome 6q. We explore whether schizophrenia and affective disorder have common susceptibility gene on 6q in Han Chinese population.

**Methods:**

In the present study, we genotyped 45 family trios from Han Chinese population with mixed family history of schizophrenia and affective disorder. Twelve short tandem repeat (STRs) markers were selected, which covered 102.19 cM on chromosome 6q with average spacing 9.29 cM and heterozygosity 0.78. The transmission disequilibrium test (TDT) was performed to search for susceptibility loci to schizophrenia and affective disorder.

**Results:**

The results showed STRs D6S257, D6S460, D6S1021, D6S292 and D6S1581 were associated with susceptibility to psychotic disorders. When families were grouped into schizophrenia and affective disorder group, D6S257, D6S460 and D6S1021, which map closely to the centromere of chromosome 6q, were associated with susceptibility to schizophrenia. Meanwhile, D6S1581, which maps closely to the telomere, was associated with susceptibility to affective disorder. But after correction of multiple test, all above association were changed into no significance (P > 0.05).

**Conclusion:**

These results suggest that susceptibility of schizophrenia and affective disorder not associated with loci on chromosome 6q in Han Chinese population.

## Background

The distinction between schizophrenia and affective disorder was historically based on distinct phenomenologies and long-term courses. A differential nosology and etiology was postulated, however never convincingly proven [1]. Opinions vary as to whether these disorders are etiologically distinct or represent points on a continuum of liability. Speculating from Kraepelin's view, schizophrenia does not aggregate in families of affective illness patients nor is there increased incidence of mood disorder in relatives of chronic schizophrenics. However, Rudin found risks of suffering from schizophrenia and mood disorder did not differ significantly among schizophrenic sibs [2]. Subsequently investigators found an increased risk of schizophrenic spectrum disorders among the first-degree relatives of probands with a family history of major mood disorders [3]. Conversely, relatives of probands with a family history of schizophrenic spectrum disorders were at a greater risk of affective illness than relatives of probands with no family history. Increasing evidence from molecular genetics also suggests an overlap in genetic susceptibility across the traditional classification systems. This has been suggested for linkage regions: 6q12-25, 13q32-q34 and 22q11-q22, and specific genes: DAOA(G72), DISC1, and NRG1 [4].

Several linkage studies across multiple population groups provide convergent support for susceptibility loci for schizophrenia – and, more recently, for affective disorder – on chromosome 6q. The first report of linkage findings on 6q13-26 in schizophrenia came from [5] which has accumulated support from a number of studies [6-17]. Recently, susceptibility loci to affective disorder were also reported to map to 6q [12,18-29], reviewed these results of positive linkage and association, and concluded that five regions (~91 Mb, ~113 Mb, ~126 Mb, ~133 Mb and ~162 Mb) could harbor susceptibility gene(s) to both schizophrenia and affective disorder. These phenomena lend support to the notion that affective disorder or a subset is associated with the liability to schizophrenia on chromosome 6q. In order to investigate whether schizophrenia and affective disorder have some genetic relationship on chromosome 6q, we recruited family trios with mixed family history of schizophrenia and affective disorder from the Han Chinese population for study using the transmission disequilibrium test (TDT).

## Methods

### Subjects

A mixed family was defined as one in which members suffer from both schizophrenia and affective disorder separately, namely at least one patient with schizophrenia and another patient with affective disorder among three-generation relatives. We recruited 45 family trios, composed of the probands and their biological parents, with mixed family history of schizophrenia and affective disorder. Here the probands were outpatients or inpatients from Shanghai Mental Health Center. Clinical diagnosis was made according to ICD-10; an independent clinician using the same criteria reviewed all diagnoses. Blood samples were obtained from the family trios. The investigation was carried out in accordance with the latest version of the Declaration of Helsinki, that the study design was reviewed by an appropriate ethical committee and that informed consent of the participants was obtained after the nature of the procedures had been fully explained. The probands included 21 male patients and 24 female ones, 26 diagnosed with schizophrenia and 19 with affective disorder (including 6 diagnosed with depressive disorder and 13 with single manic episode, respectively), mean age of patients were 28.7 ± 8.9 years, mean age of onset were 22.7 ± 8.0 years, mean duration were 5.9 ± 6.4 years; and the mean age of their parents were 58.5 ± 10.4 years.

### Genotyping

Genomic DNA was extracted from peripheral blood leukocytes of each subject. The short tandem repeat (STR) markers used in this study were modified from the ABI PRISM Linkage Mapping Set Version 2.5. These included 12 STRs (D6S257, D6S460, D6S462, D6S1021, D6S1698, D6S1639, D6S262, D6S292, D6S308, D6S441, D6S1581, D6S1697) on chromosome 6q, which spanned 102.19 cM with a mean interval of 9.29 cM, heterozygosity of each STR above 0.50 and average heterozygosity 0.78 (see Table 1). Markers were amplified by polymerase chain reaction (PCR) with a Gene Amp PCR System 9700 (Perkin-Elmer, Foster City, CA). Electrophoresis was performed with an ABI PRISM 3730 sequencer (Perkin-Elmer). The PCR products were genotyped with ABI GeneMapper software (Perkin-Elmer).

**Table 1:** 12 short tandem repeat (STR) markers used in this study

**Marker**	**map position (cM)**	**polymorphisms**	**heterozygosity**
D6S257	79.92	dinucleotide	0.87
D6S460	89.83	dinucleotide	0.82
D6S462	99.01	dinucleotide	0.66
D6S1021	112.20	trinucleotide	0.73
D6S1698	118.08	dinucleotide	0.82
D6S1639	124.11	dinucleotide	0.91
D6S262	130.00	dinucleotide	0.83
D6S292	136.97	dinucleotide	0.83
D6S308	144.46	dinucleotide	0.75
D6S441	154.10	dinucleotide	0.87
D6S1581	164.78	dinucleotide	0.72
D6S1697	182.11	dinucleotide	0.57

*12 STR markers are listed in turn according to map position. the polymorphism of D6S1021 is trinucleotide repeat, other markers are dinucleotide repeat. Heterozygosity of polymorphisms is between 0.57~0.91.

### Statistical analysis

Hardy-Weinberg equilibrium and statistical differences in genotype and allele frequencies between probands and parents were evaluated using the χ^2 ^test at a significance level of 0.05. Family-based association analyses was performed with applying the transmission disequilibrium test (TDT), where preferential allelic transmission from heterozygous parents to affected offspring is tested by applying (b-c)^2^/(b+c) statistics (Mc Nemar's equation) and χ^2 ^test.

## Results

The genotype distributions of total markers in the patient group and parent group did not deviate significantly from Hardy-Weinberg equilibrium in the patient or parent group (*P *> 0.05). Taking all family trios as study subjects, we found five STRs D6S257 at 79.92 cM, D6S460 at 89.83 cM, D6S1021 at 112.20 cM, D6S292 at 136.97 cM and D6S1581 at 164.78 cM were associated with susceptibility to psychotic disorders (see table 2). Then the family trios were grouped into schizophrenia group and affective disorder group according to diagnoses of probands and analyzed separately by TDT. We found that D6S257, D6S460 and D6S1021, which map closely to the centromere of chromosome 6q, were associated with susceptibility to schizophrenia. Meanwhile, D6S1581, which maps closely to the telomere, was associated with susceptibility to affective disorder. But after correction of multiple test, all above association were changed into no significance (*P *> 0.05).

**Table 2:** Transmission disequilibrium test (TDT) in total family trios

**Marker**	**Allele**	**Transmitted**	**Non-transmitted**	**χ**^**2**^	**p-val**
D6S257	173	0	4	4.00	0.046
D6S460	283	14	4	5.56	0.018
D6S460	287	2	9	4.45	0.035
D6S1021	134	0	4	4.00	0.046
D6S292	166	5	14	4.26	0.039
D6S1581	273	29	13	6.10	0.014

* Markers are Listed in table when *p*-val < 0.05.

## Discussion

Heretofore the pathogenesis of schizophrenia and affective disorder have been unclear. Evidence from family, twin and adoption studies indicate that both genetic and environmental factors are involved in the etiology of these diseases. Molecular genetics studies suggest that they may be heterogenous and polygenic diseases. Despite the widely accepted view that schizophrenia and affective disorder represent independent illnesses and have different modes of inheritance, some data in the literature suggest that these diseases may share some genetic susceptibility. Many linkage analyses have suggested that the chromosome 6q region could harbor susceptibility loci to schizophrenia. Recently loci for affective disorder were reported to map in the 6q region. These results suggest that the relationship between chromosome 6q and susceptibility to schizophrenia or affective disorder deserves further study. Craddock et al [4] by meta-analysis and Kohn et al [29] by a topographic approach reviewed all reported results, and both concluded similarly that susceptibility genes to schizophrenia and affective disorder may both be located on chromosome 6q. Ewald et al [19] and Dick et al [20] reported positive linkage between D6S1021 and susceptibility to affective disorder.

The present study was performed in mixed pedigrees for schizophrenia and affective disorder to investigate whether two diseases share common genetic loci on 6q. Though primary results showed STRs D6S257, D6S460, D6S1021, D6S292 and D6S1581 were associated with susceptibility to psychotic disorders. Further grouped analysis showed that D6S257, D6S460 and D6S1021 were associated with susceptibility to schizophrenia, D6S1581 associated with susceptibility to affective disorder. But after correction of multiple tests, there is no significance association between loci on 6q and susceptibility of psychiatric disorders, including schizophrenia or affective disorder. These results suggest that susceptibility of schizophrenia and affective disorder not associated with loci on chromosome 6q in Han Chinese population. One of most limitations for the study is small sample size because of the mixed family trios with schizophrenia and affective disorders correspondingly too mall. For the reason, we did not differentiate the mean age of patients, mean age at onset, mean duration, etc. for both disorders to analyze. This and differences in population, allelic and locus heterogeneity may explain our inability to replicate some previous results. Future study is needed to collect a larger number of better-characterized trios in different population and avoid false negative association.

## Conclusion

These results suggest that susceptibility of schizophrenia and affective disorder not associated with loci on chromosome 6q in our family trios of Han Chinese population.

## Competing interests

The author(s) declare that they have no competing interests.

## Authors' contributions

Dr. Zuowei Wang and Prof. Yiru Fang participated in the design of the study and drafting the manuscript. Dr. Zuowei and Dr. Shunying Yu carried out the molecular genetic studies, participated in the sequence alignment and performed the statistical analysis. Dr. ChengMei Yuan Dr. Wu Hong, Dr. Zhenghui Yi, Prof. Sanduo Jiang and Prof. Zucheng Wang participated in sample collection. Prof. John R. Kelsoe helped to review the manuscript. All authors read and approved the final manuscript.
